# Supplementary material for: Uncovering common disease mechanisms and critical biomarkers in Crohn’s disease with concurrent psoriasis and exploring potential therapeutic agents
Source: PLoS One. 2025 Jun 20;20(6):e0324007. doi: 10.1371/journal.pone.0324007 (PMC12180644; doi:10.1371/journal.pone.0324007)
Supplement: Attachment — Submitted filename: Response to Reviewers.docx [file pone.0324007.s002.docx]

**Response to Reviewers**

**Manuscript ID:** PONE-D-24-43295

**Title:** Uncovering Common Disease Mechanisms and Critical Biomarkers in Crohn’s Disease with Concurrent Psoriasis and Exploring Potential Therapeutic Agents

**Deer editors and reviewers,**

We thank all the editors and reviewers for your valuable comments and suggestions. We have carefully revised the manuscript to enhance its clarity and facilitate the understanding of the readers. Our point-to-point responses are presented in the following. We hope that the revision would satisfactorily address the comments and concerns of the editors and reviewers.

**Style requirements**

We have revised the style of the manuscript according to the requirements of journal.

**Acknowledgments**

We have removed the fund support mentioned in the acknowledgements.

**Financial disclosure**

We have modified it to “This work was supported by the National Natural Science Foundation (82000528). The funders had no role in study design, data collection and analysis, decision to publish, or preparation of the manuscript.”

**Data Availability Statement**

We have modified it to “This study utilized the following publicly available gene expression datasets: GSE95095, GSE13355, GSE102133, and GSE14905. These datasets can be queried and downloaded from the Gene Expression Omnibus (GEO) database (https://www.ncbi.nlm.nih.gov/geo/) using the accession numbers mentioned above.”

**Reviewer #1**

**Comments:**

“These are very interesting research results. The research method used is appropriate, using an exclusive analysis method. The results obtained are reasonable and very interesting. The discussion items are also very interesting data and discussions, although the contents of the discussions are very diverse due to the exclusive analysis. There are no particularly inappropriate parts or additional questions.”

**Response:**

We thank the reviewer for recognizing the technical validity of our work. All conclusions have been carefully re-examined to ensure they strictly align with the presented data.

**Reviewer #2**

**Major Comments**

**Comment #1:**

“It is unclear whether the psoriasis dataset includes patients with concurrent Crohn’s disease and vice versa. If multiple patients in each dataset have both diseases, this could introduce bias in the hub gene analysis results. Clarification on this point is necessary.”

**Response #1:**

We confirm that the original datasets (GSE95095 and GSE13355) do not specify comorbid status. This study focuses on shared mechanisms between the two diseases. We have added this limitation in the Discussion and recommended future studies to validate findings in comorbid cohorts (Pages 24-25, Lines 528-545).

**Comment #2:**

“To enhance clinical validation, the expression levels of the identified hub genes should be confirmed in patients who have both Crohn’s disease and psoriasis.”

**Response #2:**

We fully agree that clinical validation in comorbid patients would strengthen our findings. However, due to the scarcity of publicly available transcriptomic datasets for CD-PSO comorbidity and ethical barriers to recruiting new comorbid cases within the revision timeline, direct validation was unfeasible. To address this, we:

Validated hub gene diagnostic value in independent CD (GSE102133) and Psoriasis (GSE14905) cohorts.

Added literature evidence linking these genes to neutrophil pathways in both diseases (Pages 22-23, Lines 465-491).

Explicitly stated this limitation and proposed future validation in the Discussion (Pages 24-25, Lines 528-545).

**Comment #3:**

“While the study mentions a 9.6% prevalence of psoriasis in Crohn’s disease, there is no discussion on the prevalence of Crohn’s disease among psoriasis patients. A bidirectional approach is essential when examining the relationship between these two diseases. Additional discussion in the Introduction and Discussion sections is recommended.”

**Response #3:**

We have expanded the Introduction with new epidemiological data:

“Patients with psoriasis are 1.70 times more likely to develop CD” (Page 4, Line 77).

**Comment #4:**

“Psoriasis comprises multiple subtypes, including psoriasis vulgaris, psoriatic arthritis, and pustular psoriasis, each associated with distinct genetic polymorphisms. It is important to clarify which specific subtype(s) are represented in the psoriasis dataset and whether a similar classification exists for Crohn’s disease in the dataset used.”

**Response #4:**

We carefully reviewed all publicly available datasets of Crohn's disease and psoriasis used in this study. In the description of the data sources, none of the datasets mentioned specific subtypes of Crohn's disease or specific subtypes of psoriasis (such as psoriasis vulgaris, psoriatic arthritis, pustular psoriasis, etc.). We have pointed out the limitations of the paper in our discussion. Suggestions for future research can be further validated through subtype stratification analysis(Pages 24-25, Lines 528-545).

**Comment #5:**

“The identified hub genes are primarily associated with general immune and inflammatory responses. However, there is insufficient discussion on why these specific genes are particularly relevant in the comorbidity of Crohn’s disease and psoriasis. A deeper mechanistic analysis and discussion would strengthen the novelty of the findings.”

**Response #5:**

We have added a new mechanistic hypothesis in Discussion(Pages 22-23, Lines 465-491).

**Minor Comment**

**Comment:**

“In the Abstract, the abbreviation PPI (Protein-Protein Interaction) appears for the first time without explanation. A brief description should be provided to ensure clarity for readers.”

**Response:**

Revised to "protein-protein interaction (PPI) network" (Page 2, Line 33).

We tried our best to improve the manuscript and made some changes marked yellow in revised paper which will not influence the content and framework of the paper. We appreciate for editors and reviewers’ warm work earnestly, and hope the correction will meet with approval. Once again, thank you very much for your comments and suggestions. If you need further information or have any other suggestions, please feel free to let us know and we will fully cooperate.

Best regards!
